# P09-01 Prevalence and determinants of physical activity among schoolchildren in Abidjan-Côte d'Ivoire

**DOI:** 10.1093/eurpub/ckac095.131

**Published:** 2022-08-29

**Authors:** Julie-Ghislaine Sackou-Kouakou

**Affiliations:** Félix Houphouët Boigny University, ABIDJAN, Côte d'Ivoire

**Keywords:** Physical activity, health promotion, schoolchildren

## Abstract

**Background:**

The health benefits of a physically active lifestyle during adolescence include improved muscular fitness, bone and cardiometabolic health, and positive effects on weight. Physical activity has also a positive impact on cognitive development and socializing. Trends show a decline in its practice, especially in developing countries. As the potential related-factors of physical activity are not commonly assessed in Ivorian youth, we investigated factors associated with physical activity among schoolchildren in Abidjan.

**Methods:**

A cross-sectional study was carried out in a random sample of 394 schoolchildren aged 11-20 years in 2019, in Abidjan, Southern Côte d'Ivoire. Moderate-to-vigorous physical activity was assessed using a questionnaire based on Youth Risk Behaviour Surveillance System (YRBSS). Height and weight were objectively measured and body mass index (BMI) was calculated. Demographic and socioeconomic characteristics and dietary factors were also collected. Chi square test was used to compare proportions.

**Results:**

The majority of children (71.3%) do not achieve the daily recommended duration of physical activity (at least 60 minutes/day). Walking to school (80.4%) was the most common physical activity. Schoolchildren that were male (p = 0.039), had normal BMI (p = 0.04), consumed water during meals (p = 0.000) were the more physically active. The others factors that increase physical activity in our population were physically active mothers (p = 0.037) and the presence of sports facilities not far from their home (p = 0.011). Socioeconomic status was unrelated to physical activity.

**Conclusions:**

More actions or opportunities are needed to increase physical activity of schoolchildren (especially girls). It is necessary to install sports facilities that are easily accessible to them. Illustrations of parents are also important.

